# Triglycerides as Novel Phase-Change Materials: A Review and Assessment of Their Thermal Properties

**DOI:** 10.3390/molecules25235572

**Published:** 2020-11-27

**Authors:** Rebecca Ravotti, Jörg Worlitschek, Colin R. Pulham, Anastasia Stamatiou

**Affiliations:** 1 Competence Centre Thermal Energy Storage (TES), Lucerne University of Applied Sciences and Arts, 6048 Horw, Switzerland; joerg.worlitschek@hslu.ch (J.W.); anastasia.stamatiou@hslu.ch (A.S.); 2 EaStCHEM, School of Chemistry, The University of Edinburgh, Edinburgh EH9 3FJ, UK; c.r.pulham@ed.ac.uk

**Keywords:** triglycerides, thermal energy storage, phase change materials, thermal properties, enthalpy, melting point, latent heat storage, PCM, LHS, TES

## Abstract

Latent Heat Storage (LHS) with Phase-Change Materials (PCMs) represents a high energy density storage technology which could be applied in a variety of applications such as waste heat recovery and integration of renewable energy technologies in energy systems. To increase the sustainability of these storage solutions, PCMs have to be developed with particular regard to bio-origin and biodegradability. Triglycerides represent an interesting class of esters as the main constituents of animal and vegetable fats, with attractive thermal properties. In order to be used as PCMs, the thermal behaviour of triglycerides has to be fully understood, as in some cases they have been reported to show polymorphism and supercooling. This study assesses the suitability of triglycerides as PCMs by reviewing the literature published so far on their behaviour and properties. In particular, melting points, enthalpies of fusion, polymorphism, thermal conductivities, heat capacities and thermal cycling stabilities are considered, with a focus on LHS and thermal energy storage applications. In addition, the efforts conducted regarding modelling and the prediction of melting points and enthalpies based on chemical structures are summarized and assessed.

## 1. Introduction

Latent Heat Storage (LHS) systems using Phase-Change Materials (PCM) as heat-storage media are known to be advantageous due to their high energy densities and ability to charge and discharge at constant temperatures. As such, the development of LHS systems can contribute to meeting the future energy requirements and reaching the carbon-footprint reduction goals set for the upcoming years [1,2,3,4]. To ensure the effective development of LHS, first the appropriate PCM for the application of interest must be selected. In order to be considered suitable for LHS applications, PCMs should possess the following properties:

- high enthalpy of fusion;
- high thermal conductivity;
- narrow phase-change transition temperature;
- high thermal stability;
- low toxicity;
- low flammability.

In additional, ideal PCMs should be bio-based, sustainable and readily available from renewable feedstocks [5,6].

**Figure 1:**
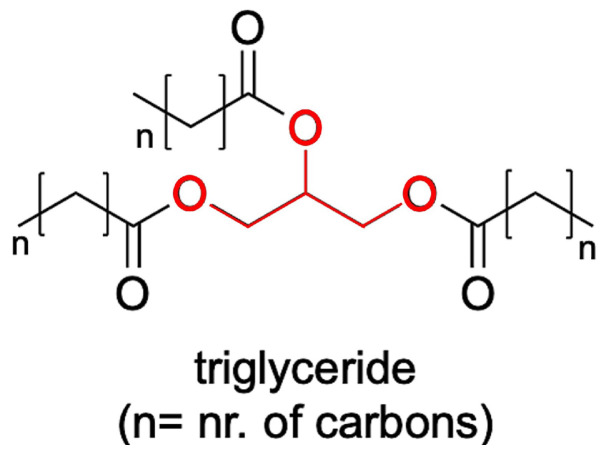
Typical structure of a triglyceride. Triglycerides are formed from a molecule of glycerol (middle, red) and three of fatty acids (side chains, black). The letter “n” represent an unspecified number of carbons in the chains.

Several classes of materials have been investigated, but triglycerides have often been overlooked despite their wide occurrence in nature and their attractive properties. Triglycerides are esters derived from glycerol and three fatty acid molecules, and are the main constituents of animal and vegetable fats. Their typical structure is shown in Figure 1.

As such, they act as natural energy storage components in the human body and other species [7]. At present, triglycerides are widely used in the food formulation industry and as precursors for the production of bio-diesel components and glycerin. Some disadvantages presented by triglycerides have halted their development as PCMs. The main one is polymorphism, or the tendency to crystallize in different solid-state structures, each with their own characteristic phase-change transition and enthalpies [8]. However, the thermal properties and behaviour of triglycerides have been widely studied over years, and have been shown to offer great potential as PCMs, provided that polymorphism is avoided and narrow and reliable phase-change transition temperatures are achieved.

The present review aims at assessing the suitability of triglycerides as possible PCMs for LHS applications. To do so, all published thermal data on saturated, unsaturated and asymmetrical triglycerides have been collected and reported hereby. In particular, the most relevant properties for thermal energy storage applications are evaluated, namely melting point, enthalpy of fusion, heat capacity and thermal conductivity. Due to the vast variety of glycerides, the thermal properties of mixtures of triglycerides and of mono- or diglycerides (derived by glycerol and one or two fatty acid chains, respectively) have not been considered for the study presented hereby.

As the polymorphic behaviour and the crystalline arrangement of triglycerides plays a major role on their thermal behaviour, an overview of their known polymorphic transformation is also given.

To this aim, this review is organized as follows:

- overview on the polymorphism of triglycerides and known crystalline arrangements;
- summary of triglycerides’ thermal properties and considerations on trends correlating them to the chemical structures;
- overview of modelling methods employed to predict the thermal properties;
- evaluation of studies conducted on triglycerides as PCMs;
- conclusions and outlook for thermal energy storage applications.

This is the first review on triglycerides to include the topic of their experimental and predicted thermal properties as well as their polymorphic behaviour, and associated applications for thermal energy storage (TES).

## 2. Polymorphism of Triglycerides

Triglycerides are characterized by a typical tendency to crystallyze as polymorphs, meaning that they are able to form different crystalline structures depending on several factors that include: chemical composition, presence of impurities, thermal history and measurement conditions (such as the heating or cooling rate in dynamic conditions), and the dwell time under isothermal conditions.

**Figure 2:**
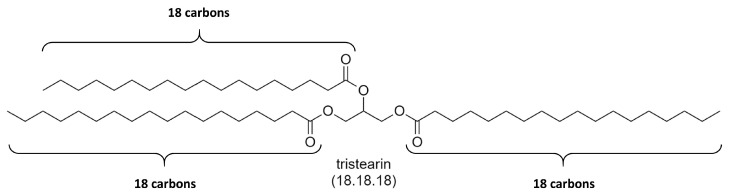
Chemical structure of Tristearin. Since tristearin is formed from glycerol and three molecules of stearic acid (18 carbons in alkylic chain), tristearin is abbreviated as 18.18.18.

The polymorphism of triglycerides has been the subject of intensive study since 1853 when Duffy [9] noticed the occurrence of three melting points in tristearin at 52 ${}^{\circ }$C, 64.2 ${}^{\circ }$C and 69.7 ${}^{\circ }$C. The chemical structure of tristearin is shown below in Figure 2.

In particular, he noticed that tristearin was able to adopt different forms depending on the thermal conditions in which it was melted or crystallized, and that with higher degrees of impurity the second melting point could no longer be detected. Therefore, he proposed that molecules of tristearin could arrange themselves to form distinct crystalline structures, thereby giving rise to polymorphism.

However, it was not until the early 1900s with the advent of X-ray diffraction methods that the existence of different crystal structures could be confirmed. Following the introduction of this technique, Duffy’s findings inspired researchers to investigate more intensively the polymorphism of triglycerides, causing a surge in the number of publications related to the subject between 1939 and 1983 [10,11,12,13,14,15,16,17,18,19].

Based on early X-ray diffraction studies, the polymorphic behaviour of saturated symmetrical triglycerides (particularly trilaurin, trimyristin, tripalmitin and tristearin) have been studied to identify four distinct polymorphic phases. These are namely, listed in order of growing melting temperature and stability, the γ, α, β,’ and β forms. The γ phase was denoted by Malkin [11] as similar to an anisotropic glass, thus lacking order and symmetry, and was clearly distinguished from the other phases as the “vitreous phase”. However, later the vitreous phase has been recognized and officially referred to as the α phase, while the α phase originally described by Malkin has now been identified as one of the possible β′ forms. Therefore, at present, the official convention for the determination of triglycerides’ polymorphic phases follows Lutton’s formulation [20]:

- α form: it appears as an amorphous mass of small crystals with molecules organized in random degrees of rotation around the chain axis but at constant distances [21,22]. For simple triglycerides, this corresponds to hexagonal subcells [23]. The α phase is similar to a “colloidal” glass [11]. It is thermodynamically unstable, but kinetically favorable; therefore, it will be the prevalent phase in fast heating-cooling conditions. This form usually exhibits no alternation in physical properties for odd and even number of carbon atoms. Only one α form exists for a given compound [15,24]. Over time, the α phase will crystallize irreversibly to form the β phase.
- β′ form: it is usually consisting of spherulitic crystals [21,22]. For simple triglycerides, this corresponds to orthorombic subcells [23]. It is thermodynamically unstable with melting points between those of the α and β phases. Mixtures, asymmetrical or unsaturated triglycerides are more prone to presenting β′ phases. As reported by Hoerr et al. [14] the β′ phase is highly influenced by the presence of impurities, as percentages over 2–3% can block completely its formation. Different variations of the β form can exist for given compounds [15,24]. This form is the most difficult to characterize, as it is the most unstable phase and will spontaneously and irreversibly evolve into β phase over time.
- β form: it is easily detected as it forms needle-like crystals with the molecules locked in a definite position, and the hydrocarbon chains tilted at an angle of circa 65 ${}^{\circ }$C to the end-group planes. For simple triglycerides, this corresponds to triclinic subcells [23]. It is the most thermodynamically stable form and as such will be favoured in slow heating-cooling conditions. The β phase shows alternation between odd and even number of carbon atoms in a series, and more than one form for given compound are reported [15,24]. Due to their highly ordered state, β crystals cannot be spontaneously formed from an α, β′ or β melt in the absence of thermal history, but will rather grow from existing α or β′ crystals, or directly from a β melt if a thermal history or seed crystals are present.

**Figure 3:**
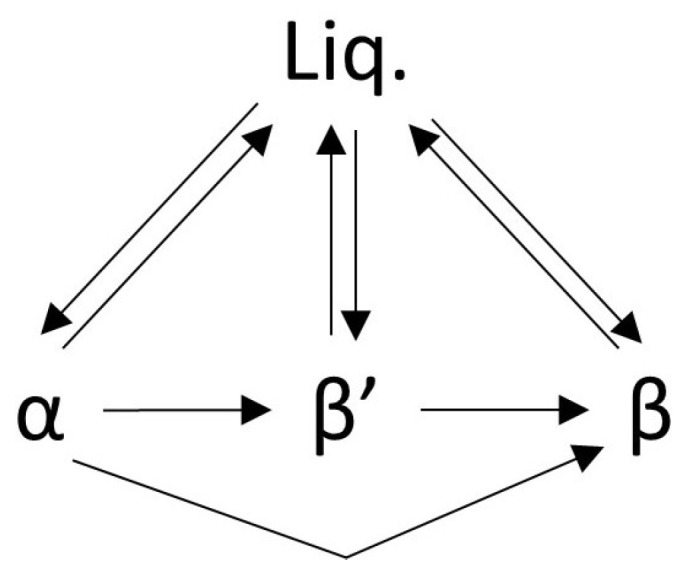
Scheme summarizing the relation between each polymorphic form of triglycerides. Each phase can be reversibly formed from the melt, however the transition from α→β′→β or from α→β is irreversible. Image reproduced from Hernqvist [23].

Figure 3 shows the relation between the different polymorphic phases. This has first been theorized by Malkin [11] from both thermal and powder diffraction data of a series of saturated di- and triglycerides (namely 1,2-dipalmitin, 1,2-dilaurin, tripalmitin, 2-myristodipalmitin, 2-palmitodimyristin, 2-oleodistearin). This has been later assumed to be valid for all triglycerides [23]. To summarize, since the α phase is kinetically stable, α crystals can reversibly form from the melt (liq.) in fast heating-cooling conditions, whereas β′ and β crystals can only form irreversibly from pre-existing crystals [25], or in extremely slow heating-cooling conditions from the corresponding melt. Thus, one option to maintain the β form constant is to include seeds in the melt to promote nucleation [25]. Alternatively, the possibility of creating the β′ and β form directly from solvents is also discussed in the literature [17,23]. Interestingly, once formed, due to the triglycerides’ ability to maintain a thermal “memory” of past crystalline forms, it is theorized the β crystals will not revert to less stable phases upon cycling unless the thermal history is erased via high temperature treatments [26,27,28]. The β′ phase is the hardest to characterize as it is metastable, thus, it tends to transform directly to β over time whenever possible [10,13,14]. Additionally, the passage from α directly to β can occur without the β′ taking place. However, it must be noted that the β′ phase occurs more often and is most stable in the presence of unsaturation or asymmetrical molecules.

**Figure 4:**
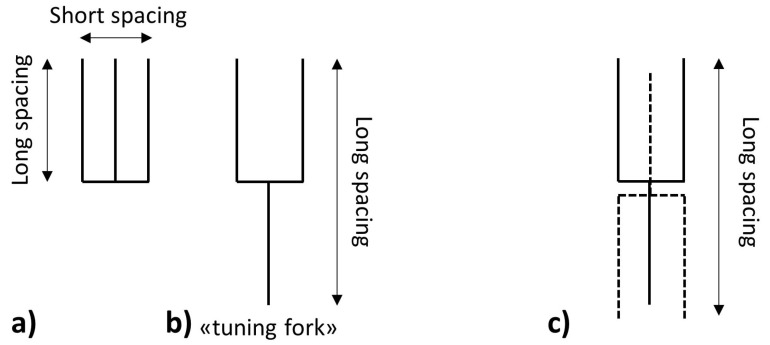
(**a**) The originally proposed arrangement of triglyceride molecules in the crystal lattice; (**b**) the new “tuning fork” structure proposed by Malkin [11] which explains the increase in long spacings measured. (**c**) The orientation of two molecules in the “tuning fork” shape in a crystal lattice. Images reproduced from Malkin [11].

Based on the data obtained from the analysis of long and short d-spacings in powder XRD patterns, first Malkin [11] and Lutton [15], then later Hagemann [18] and Hernqvist [23], described the structural arrangements of the molecules in the lattices which are still widely accepted nowadays. In particular, given that the long d-spacings are too large to be representative of the length of one single acid chain, the so-called “tuning fork” structure was proposed, which explains why the values match twice the length of the acid chain (Figure 4a). In general, the molecules are thought to assume an arrangement “head to tail” as shown in Figure 4b.

**Figure 5:**
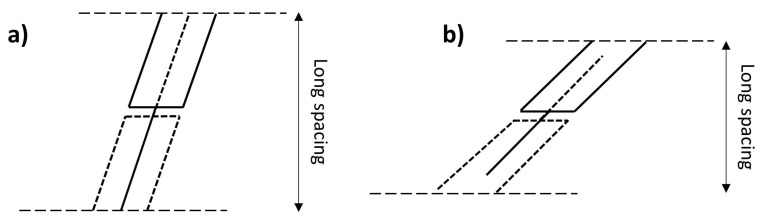
The tilting of the aliphatic chains in the β′ (**a**) and β (**b**) form as proposed by Malkin [11] and Lutton [20], which rationalises the different long d-spacings measured for the polymorphic phases. Images reproduced from Malkin [11].

However, depending on the crystalline structure, different degrees of tilting occur, which cause changes in the long d-spacings. Lutton [20] reported the long d-spacing of tristearin and observed a progressive decrease in their values from 50.6 to 46.8 and 45.2 Å corresponding to the α-, β′- and β-phases, respectively. This reflects an increasing degree of tilting of the hydrocarbon chains plane compared to the end groups axis (Figure 5a,b).

In terms of short d-spacings, what allows us to distinguish between the various crystal phases is the number of peaks measured. That is, given the higher degree of symmetry when progressing from α→β′→β phase, an increasing number of short spacing is observed. For example, Lutton [20] reports for both tripalmitin and tristearin only one strong peak at about 4.14 Å for the α phase corresponding to the intramolecular distance between aliphatic chains. In the case of the β′-phase two peaks are observed at 4.18 and 3.78 Å and for the β-phase three peaks arise from the triclinic unit cell at 4.60, 3.85 and 3.70 Å [29].

**Figure 6:**
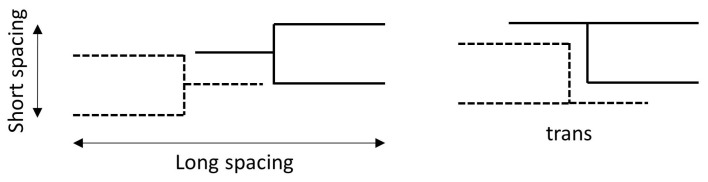
The different arrangements of asymmetrical or unsaturated triglycerides in the crystal lattice as proposed by Malkin [11] and Hernqvist [23]. Images reproduced from Malkin [11] and Hernqvist [23].

What is reported above is true for symmetrical and saturated triglycerides. In the case of unsaturated or asymmetrical triglycerides, different conformations arise. Malkin [11] and Hernqvist [23], suggested that in this case the molecules arrange themselves as depicted in Figure 6, thus causing a large increase in the long and short d-spacings [10,11,20,23,30,31].

However, due to the size of triglyceride molecules and their tendency to form partially amorphous structures, growing single crystals that would confirm the theoretical structures presented above still remains a challenge.

Exhaustive tables presenting the short and long spacings measured using XRD for each compound are presented in the literature and can be retrieved from the references mentioned above. However, as this is not the main focus of this study, the XRD data will not be discussed further in details.

## 3. Thermal Properties of Triglycerides

Several sources in the literature describe the thermal properties, especially melting points, of triglycerides [16,31,32,33,34,35,36,37,38,39,40,41]. Despite the numerous investigations conducted on this topic, discrepancies are still present and several data are lacking. Therefore, a summary is required in order to obtain a better overview on this class of materials.

In the next pages, the thermal properties collected from the literature are reported. As it is often unspecified whether the melting points reported in the literature are referring to onset or peak values, they are hereby assumed to represent peak temperatures.

The chemical structures of all compounds mentioned in the next pages are shown in Appendix A and are divided according to triglycerides’ categories, namely saturated symmetrical (SST), unsaturated symmetrical (UST) and saturated asymmetrical (SAT).

### 3.1. Saturated Symmetrical Triglycerides (SST)

Table 1 summarizes the data collected for the peak melting temperatures of SST. The SST are abbreviated according to the number of carbons in each aliphatic chain as shown in Table 2. For example, as tricaprylin is composed of 8 carbons in each aliphatic chain, and as all three chains are equal, it is abbreviated as 8.8.8).

**Figure 7:**
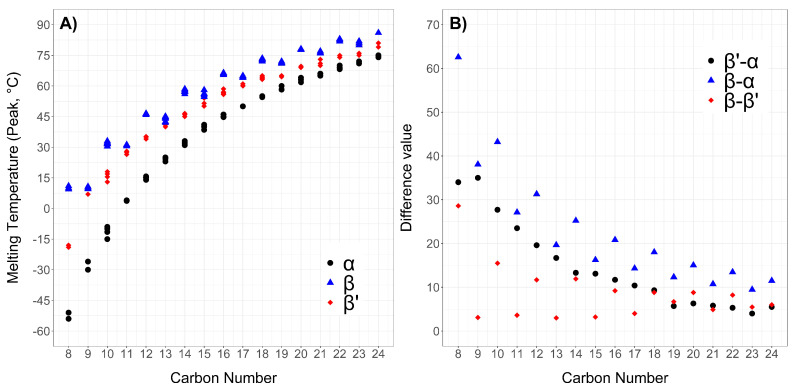
(**A**) Peak melting points ( ${}^{\circ }$C) collected in literature of the α (black, round), β′ (red, diamond) and β (blue, triangle) phases of saturated symmetrical triglycerides plotted against the carbon number of the precursor fatty acids (between 8 and 24). A clear alternation between odd- and even-numbered triglycerides can be observed for the β phases, while it is absent in the α and barely noticeable in the β′ phase. (**B**) Difference between the average melting points of the polymorphic phases plotted against the carbon number of the precursor fatty acids (between 8 and 24). A clear reduction in the differences between the melting points of each phase can be seen for increasing carbon numbers, indicating that the polymorphs have more similar behaviour to each other for longer triglycerides.

The SST melting peaks range from sub-zero values, for example, −54 ${}^{\circ }$C for the α-phase of tricaprylin, to 93 ${}^{\circ }$C for the β′ phase of trimelissin (or 30.30.30). The data collected from the various sources are in good agreement, with variations mostly ≤3 ${}^{\circ }$C, despite the studies taking place at a distance of several years from one another (Figure 7A). The melting transitions of the various triglycerides generally increase in an approximately linear manner with increasing number of carbon atoms. It is yet unclear whether there is tendency to reach a plateau at temperatures near 80 ${}^{\circ }$C given the scarce data for triglycerides with carbon number ≥18.

According to Hagemann et al. [18], depending on their packing arrangements, different modifications of each polymorphic phase can arise, which cause multi-peaks profiles in the region of interest. These variations are often referred to as “submodifications”. However, no confirmation of these “submodifications” exists from XRD data. These subtransitions are reported in Table 1 with numbers in brackets next to the melting point of reference. For instance, the number (1) next to the melting point of a β′ phase indicates the melting point of the β′(1) “submodification”. Overall in the case of SST, up to two “submodifications” for the β′ phases are observed. This is in accordance with what reported by Malkin [24] and Lutton [15] as well.

Interestingly, a significant difference between the melting temperatures of the polymorphic phases could be observed for all triglycerides, in particular between the α- and β′-phases. In this regards a trend can be noticed. As the carbon number increases, the difference between the melting temperatures of the α-, β′-, and β-phases decrease and the peak melting points become closer in value to each other (Figure 7B). No mention of such a trend have been found in the literature, but one possible explanation could be a reduced mobility of the longer chains in the α- and β′- phases, thus creating a more ordered phase similar to that found for the β-phase.

**Figure 8:**
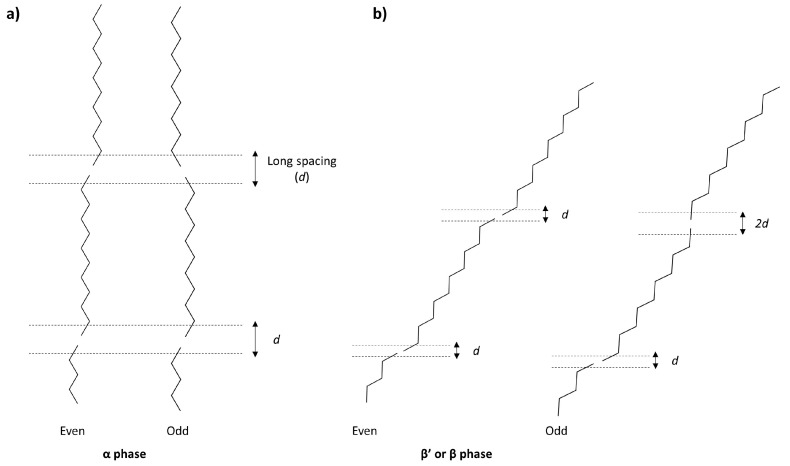
(**a**) Even- and odd-numbered aliphatic chains in the α phase. The absence of tilting in respect to the end-group plane in this polymorph generates no change in the long spacings (d) between even- and odd-numbered molecules. (**b**) Even- and odd-numbered aliphatic chains in β′ or β phases, where tilting occurs. Here, the tilting of the plane causes an enlargement in the long spacings of odd-numbered chains. Images reproduced from Malkin [24].

Another trend observed for SST is the alternation in the melting points of the β- and β′-phases of triglycerides derived from odd-numbered or even-numbered fatty acids (Figure 7A). This behaviour has previously been reported by Malkin [24] and Lutton [15] and is also reflected in the variation between the melting points of the crystalline phases shown in in Figure 7B. Boese et al. [42,43] and Ravotti et al. [44] have also reported this for alkanes and saturated fatty esters. The reason behind such alternation in the melting points is attributed to odd-numbered carbon chains’ lower degree of symmetry and less optimal intermolecular interactions compared to even-numbered chains. However, this effect is only clearly visible for the β-phase, whereas for the β′-phase it is negligible, and it is completely absent in the α-phase, where melting points are only seen increasing with increasing carbon numbers. Malkin [24] attributed this behaviour to a change in the long d-spacings in tilted odd-numbered molecules (Figure 8). As can be seen, due to a lack of tilting of the molecules in the α-phase, the long spacings result affected only in the β′ and β phase. In particular, a greater tilt of approximately 65${}^{\circ }$ occurs in the β phase.

Compared to the melting points, very limited data are available concerning the enthalpies of fusion of SST. Several studies have focused on the temperatures at which the phase change occurs, however they do not mention the phase change enthalpy. Furthermore, there is considerable variation in the reported values, reflecting the lack of precision and variability of experimental measurements. It is generally unclear how the enthalpies of fusion are obtained when polymorphism is apparent, and in some cases there is no mention of which polymorph is being studied [29]. This clearly emphasizes the need for additional, systematic investigations on this class of materials. Nevertheless, some trends can be observed for these SSTs.

**Figure 9:**
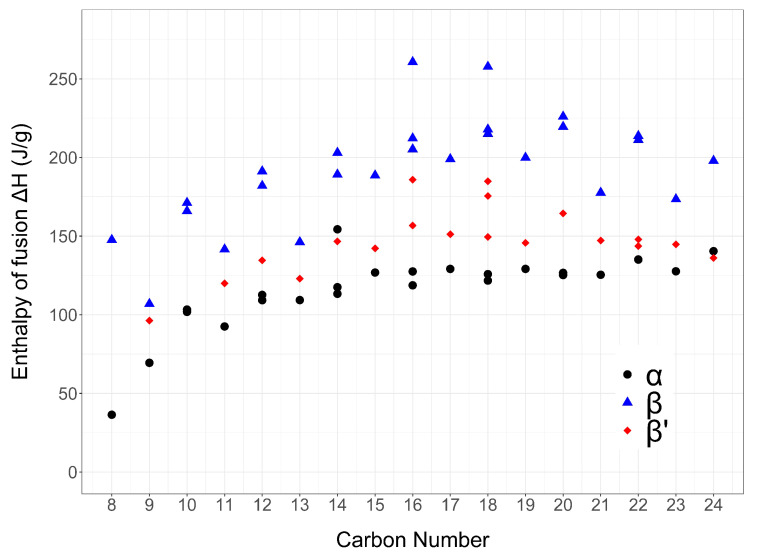
Enthalpies of fusion [J/g] collected in literature of the α (black, round), β′ (red, diamond) and β (blue, triangle) phases of saturated symmetrical triglycerides plotted against the carbon number of the precursor fatty acids (between 8 and 24). Similarly to what observed for the melting points, an alternation between odd- and even-numbered triglycerides can be noticed for all polymorphic phases.

In general, the enthalpy of fusion (Δ*H*) increases with increasing numbers of carbon atoms and differs significantly between different polymorphs (Figure 9).

A clear alternation between the properties of odd-even numbered chains is reflected in the enthalpies of fusion; those of odd-numbered chains are consistently lower than their even-numbered counterparts. This also appears for the β polymorph, while due to lack of additional data it is unclear whether it emerges for β′ and α polymorphs. Altough the odd-even alternation for enthalpies of fusion is not mentioned by Malkin [24] and Lutton [15], it has been observed by Ravotti et al. [44] in the case of saturated fatty esters. The specific causes behind this behaviour are not clear, but it could potentially be due to the lower degree of symmetry present in odd-numbered molecules.

Nevertheless, the presence of missing or incomplete data in the case of enthalpies of fusion makes it difficult to draw general conclusions on possible trends.

### 3.2. Unsaturated Symmetrical Triglycerides (UST)

**Figure 10:**
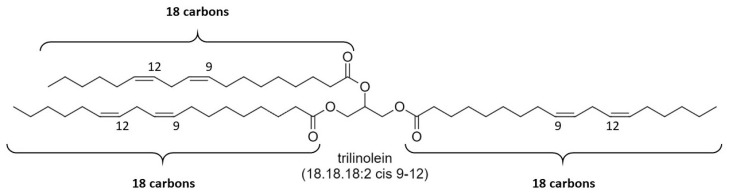
Chemical structure of trilinolein. Since trilinolein presents 2 cis double bonds at position 9 and 12 of each alkyl chain, tristearin is abbreviated as 18.18.18:2 9-12.

Table 2 summarizes the data collected for the peak melting temperatures of UST. The UST are abbreviated in a similar way to SST, but with additional information on the number and location of the unsaturation. For example, trilinolein is derived from glycerol and three linoleic acid molecules, whose carbon number is 18 and that have 2 double bonds in the cis configuration at the carbon numbers 9 and 12. Therefore, trilinolein is indicated as 18.18.18:2 cis 9-12 (Figure 10).

The studies conducted on unsaturated triglycerides are far less extensive than they are for saturated triglycerides. Several data are still missing, especially with regard to the enthalpies of fusion. Most data collected concerns the main constituents of olive oil and vegetable oils in general, such as triolein and trilinolein. Additionally, no data could be found for most of the odd-numbered UST.

Although it is difficult to draw conclusions given the lack of data, some broad observations can be made. In general, compared to SST of equal chain length, UST show lower melting points and enthalpies of fusion. Similarly to what was observed for SST, the highest phase change enthalpy seems to arise for the 18 carbon-numbered compound, tripetroselaidin (18.18.18:1 trans 6), with 179.9 J/g. The reasons behind the lower enthalpy of fusion are unclear, but could be due to an easier disruption of the crystal lattice caused by the presence of unsaturated bonds brought on by a lower degree of symmetry.

**Figure 11:**
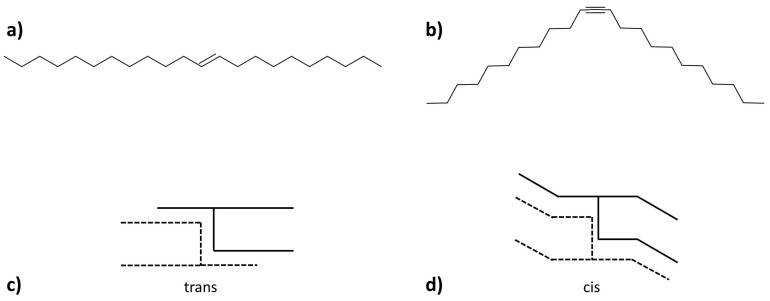
(**a**) Aliphatic chain with unsaturation in the trans configuration, meaning with the two carbon chains on opposite sides of the double bond. (**b**) Aliphatic chain with unsaturation in the cis configuration, meaning with the two carbon chains on the same side of the double bond. (**c**) Configuration proposed for the trans-unsaturated triglycerides. (**d**) Configuration proposed for the cis-unsaturated triglycerides. Images reproduced from Hernqvist [23] and Malkin [24].

While UST with double bonds in the trans configuration still present similar characteristics to SST with melting temperatures above 0 ${}^{\circ }$C and enthalpies of fusion ≥150 J/g, most of the UST in the cis configuration show the opposite behaviour with melting temperatures below 0 ${}^{\circ }$C and enthalpies of fusion ≤150 J/g. Such difference becomes more marked in the presence of increasing number of double bonds in the cis configuration. This is due to the bend that the aliphatic chain undergoes in the presence of cis-double bonds as shown in Figure 11a–d, which contributes in generating wider long spacings and lowering the overall symmetry and crystal packing compactness [23,24].

Up to three different polymorphs arise in the β′ phase as a consequence of different arrangements [23,45], contrary to SST where only two variations were observed in the β′ phase. Similarly to Table 1, the ”submodifications” are assigned to the corresponding melting points with a number in brackets next to them in Table 2.

The fact that several variations of each polymorph takes place in the β′ instead of the β phase supports the theory reported previously, according to which the β′ phase becomes more stable in the presence of mixtures, asymmetrical or unsaturated molecules. For example, Himawan et al. [37] and MacNaughtan et al. [38] analyzed the thermal properties of both pure tripalmitin and tristearin and their mixtures, and report a greater tendency for the formation of the β rather than the β′ form in samples where one species predominates, and the opposite tendency for samples with more equal compositions. Such effect is attributed to steric factors which hinder the formation of β in mixtures. The same can be assumed to be true for unsaturated triglycerides.

### 3.3. Saturated Asymmetrical Triglycerides (SAT)

Table 3 summarizes the data collected for the peak melting temperatures of SAT. Since most of the data found in the literature concerns triglycerides derived from saturated fatty acids, due to lack of data it was decided not to focus on the asymmetrical unsaturated counterparts. Similar to what has been reported for the previous categories, few values for the enthalpies of fusion of the materials investigated were reported, whereas the data on melting points was readily available from different sources.

Asymmetrical triglycerides were divided into sub categories based on the number of carbon atoms in the precursors fatty acids (n) and their positions in the final molecule. For example, n.n+2.n indicates triglycerides of which the middle chain is longer than the side chains by 2 carbons. By contrast, n.n-2.n refers to those triglycerides formed from two aliphatic chains of the same carbon number on the extremes, and one aliphatic chain shorter by two carbon numbers in the middle. Lastly, n1.n2.n3 refers to triglycerides where aliphatic chains all differ in carbon number. The names of each SAT are abbreviated in the same manner as SSTs.

Generally, the SATs reported here show melting temperatures ranging from 0 ${}^{\circ }$C for 1,2-dicaprin-3-laurin (CCL) in the α phase to a highest of 73.6 ${}^{\circ }$C for 1-stearin-2,3-dibehenin (SBB) in the β phase.

Some trends could be observed throughout the series. N.n-2.n and n.n+2.n triglycerides derived from the same fatty acids are characterized by similar melting points, such as for example 1,3-dipalmitin-2-myristin (PMP) and 1,3-dimyristin-2-palmitin (MPM) which both melt around 60.0 ${}^{\circ }$C in the β phase. Their melting points are in between those of their SST counterparts, trimyristin and tripalmitin at 58.5 and 66.5 ${}^{\circ }$C respectively. Upon comparison between n.n+2.n and n.n.n+2 triglycerides, the latter were found to possess lower melting points for the same polymorphic phase, such as for example 1,3-dipalmitin-2-stearin (PSP) and 1,2-dipalmitin-3-stearin (PPS) which melt at 68.0 and 62.5 ${}^{\circ }$C (β phases) respectively. This is to be expected due to the lower degree of symmetry of the n.n.n+2 triglycerides. This behaviour is found to persist upon comparison with the n.n+4.n+4 subgroup too, where 1-laurin-2,3-dipalmitin (LPP) for instance has the same total carbon number of 44 as MPM, but still presents a lower melting point around 54 ${}^{\circ }$C compared to MPM’s at 60.0 ${}^{\circ }$C (β phases). Triglycerides belonging to the n1.n2.n3 subgroup appear to be characterized by comparable melting points as the n.n.n+2 compounds, but lower than for n.n+2.n for equal carbon number. For example PMP (n.n+2.n), MMP (n.n.n+2) and SCP (n1.n2.n3) are all characterized by carbon numbers equal to 44, but show melting points of 60.0, 53.5 and 54.3 ${}^{\circ }$C, respectively.

Concerning the enthalpies of fusion, generally those of SAT seem lower than those of the corresponding SST. For example, although PPS presents the highest enthalpy of the whole SAT series with 198.9 J/g in its β phase, it is still lower than the enthalpies of fusion of tripalmitin or tristearin with values of about 220 J/g in the same crystalline phase. No information on the enthalpies of fusion of n.n+4.n+4 compounds and of LCL was found.

### 3.4. Heat Capacity and Thermal Conductivity

Although most data found in the literature concern the melting points of triglycerides, recently additional studies have been conducted on other thermal properties such as the heat capacity and thermal conductivity. Nevertheless, the data often prove to be unsatisfactory, in some cases with no mention of the purity of the sample investigated or of the uncertainty in the data reported.

Data exist on the heat capacities of SST derived from fatty acids with even carbon numbers from 6 to 18, while it is non-existent for the remaining saturated triglycerides (odd and even) and for the unsaturated derivatives. Therefore, it is clear that in order to promote the introduction of triglycerides in the thermal energy storage field as novel PCMs, further studies need to be conducted on their thermal properties besides melting points and enthalpies of fusion.

Table 4 summarizes the literature data collected for even numbered SST with carbon numbers between 6 and 18 concerning heat capacities and thermal conductivities. No thermal conductivity values could be found for tricaproin. The heat capacities retrieved are comparable to those of other organic materials typically used as PCM such as paraffins, and there is good agreement between the sources with the exception of tricaprin. On the other hand, discrepancies are observed between the thermal conductivity of trimyristin reported by Bogatishcheva [46] and Sari [40].

In general, the heat capacities increase linearly with increasing carbon numbers, while no clear trend is detected between the thermal conductivities and chemical structures. Morad et al. [47] analyzed the thermal properties of both simple and mixed triglycerides, and report that no pattern between specific heat capacity and carbon number is observed for mixed triglycerides. In addition, the specific heat capacities of the mixed triglycerides are lower than those of the simple triglycerides. Overall, the thermal conductivities of the triglycerides show rather low values around 0.2 W/(m· K) in agreement with those observed for other organic compounds.

### 3.5. Thermal Degradation

Limited data are available on the thermal stability of triglycerides in terms of thermal degradation. In general, due to the similarities in the chemical structure, triglycerides behave comparably to other organic molecules such as esters [44,50]. For example, as reported by Morad et al. [47] trilaurin shows an onset degradation at 150 ${}^{\circ }$C, where its specific heat capacity drops suddenly in non-inert atmosphere. Additionally, the authors measured the onset degradation temperatures of trimyristin, tripalmitin and tristearin at 181, 184 and 187 ${}^{\circ }$C respectively, which indicates that the thermal stability of triglycerides increases with increasing carbon number and chain length. This is in accordance with results reported by Ravotti et al. for fatty esters and diesters [44,50,51].

Several studies have been conducted on the mechanisms driving the thermal degradation of triglycerides and the conditions under which this occurs. Upon investigating the thermal degradation behaviour of olive oil, which is rich in triglycerides, Vecchio et al. [52] hypothesize that the decomposition of the saturated and unsaturated carboxylic chains occurs approximately in the range 160–370 ${}^{\circ }$C, whereas in oxidative conditions above 370 ${}^{\circ }$C a fast combustion of the evolved volatile components takes place, followed by oxidation of the carbonaceous residues. This is confirmed by Lee et al. [53], who observed a second peak in the thermogravimetric (TGA) curve of coconut oil around 363 ${}^{\circ }$C corresponding to the combustion of volatile residues. Sari et al. [40] have also noted a two-step degradation mechanism when evaluating the thermal stability of trimyristin, tripalmitin and tristearin. However, in contrast to reports by Morad et al. [47], they observed the onset of degradation at 150, 170 and 180 ${}^{\circ }$C for trimyristin, tripalmitin and tristearin, respectively. According to Nawar et al. [54], under oxidative conditions triglycerides may also undergo a 6-atom-ring-closure, which was confirmed when a series of lactones were shown to form by heating milk fat. Crossley et al. [55] observed this under non-oxidative conditions as well when studying the effects of heat on tricaprin in the absence of moisture at 300 ${}^{\circ }$C. After 4 h of heating under a constant nitrogen flow, both acrolein and di-n-nonyl ketone and, to a lesser extent, the symmetrical ketones di-n-butyl, di-n-amyl, and di-n-hexyl were produced in addition to the major product, decanoic acid.

Noble et al. [56] also investigated the hydrolysis of corn oil, cottonseed oil, and lard heated at 200 ${}^{\circ }$C in the presence of moisture, and noted a preference for the hydrolysis of the shorter chain and the unsaturated acids. In addition, they observed an increase in the rate of hydrolysis with the degree of unsaturation. Therefore, unsaturated triglycerides undergo earlier onset thermal degradation.

Other thermally induced reactions which may occur under non-oxidative conditions include dehydration, decarboxylation, hydrolysis of the ester bond, double bond conjugation, polymerization, dehydrocyclization, aromatization, dehydrogenation, and degradation by carbon-carbon cleavage [54].

## 4. Modelling and Prediction of Thermal Properties

Due to the importance of the thermal properties of triglycerides in defining the final characteristics of foods rich in fat, researchers have been trying to develop models able to accurately predict such properties. In fact, being able to predict the thermophysical properties of a given chemical or to assign a certain chemical structure to the required thermal property would considerably reduce the time required by the formulation of specific foods and would greatly benefit to researchers operating in this field.

Several models have been developed over the years to identify the thermal properties of a molecule starting from its chemical structure alone. As mentioned by Moorthy et al. [57], despite the great advances made in developing models capable of predicting the thermal properties of triglycerides, research for the prediction of unsaturated triglycerides is far less developed than it is for saturated triglycerides due to the lack of data collected on their thermal properties.

In 1936, King and Garner [58] formulated a simple equation to predict the enthalpies of fusion of fatty methyl or ethyl esters with chain lengths exceeding carbon number equal to 8. Their model parameters were then corrected and implemented to improve predictions for specific triglycerides groups, such as saturated and cis unsaturated compounds, and some of their polymorphs. Hagemann and Rothfus [18] contributed to this with investigations on the packing geometries and the molecular rotation freedom.

However, up to this point the majority of the models for predicting either the enthalpy of fusion or the melting point of the different polymorphic forms relied only on the total number of carbon atoms in the three alkyl groups as input variable [28]. Therefore, often when calculating the melting point of different polymorphic forms having the same carbon number the models failed. In 1990 Wesdorp [27] introduced a new model where the position and the chain length of the three alkyl groups in the triglyceride are also taken into account. Based on the work conducted by Wesdorp, Constantinou et al. [59] introduced the group-contribution (GC) method, where aside from the position and the chain length, the class to which the triglyceride belongs is also considered. In particular, triglycerides are divided into four classes, which depend on the number of different acyl groups the triglyceride contains and their position in the chemical structure.

In the latest years, Molecular Dynamics (MD) approaches have risen to prominence as reliable methods to predict the physical properties and the arrangements of molecules in the solid and liquid state. MD methods seem to predict well those physical properties that are highly dependent on the molecular arrangement, such as the density and the viscosity. A few studied in the literature have successfully predicted these properties for triglycerides [60,61]. More recently, Pink et al. [62] attempted to predict the Δ*H* of trilaurin through molecular dynamic simulations, but found that their results were consistently underestimating the Δ*H* of the solid-liquid transition. The authors suspect this to be due to the underlining assumption that all excited states are represented by one average state. Pizzirusso et al. [63] predicted the density and melting points of tristearin and tripalmitin mixtures through a coarse-grain modelling approach and found a qualitative agreement with data published in the literature. While the density and viscosity are properties mostly dependent on the arrangement of the molecules, the melting points and Δ*H* are strongly influenced by other factors as well, such as for example the carbon number, the degree of unsaturation, the degree of rotational freedom and the level of symmetry. This could be the reason behind the low accuracy of the results obtained so far with MD on the thermal properties. mInstead, MD methods’ strength is in the prediction of the interactions between molecules, and as such have been widely employed to simulate the triglycerides’ behaviour in oil-water systems as surfactants and in emulsions, rather than to predict their thermophysical properties [64,65,66].

Despite their accuracy, MD approaches present the severe disadvantage of requiring prior extensive information on the physical properties and crystalline arrangements of the molecules.

Therefore, up to 2017, according to Moorthy et al. [57], the GC-method was still the most comprehensive resource to predict the thermochemical properties of pure triglycerides. However, it presents the disadvantage of requiring prior knowledge on the specific position of each group in the molecule. Adjustments and developments to overcome this issue are currently being made and extensive research on the topic is being performed.

## 5. Triglycerides as PCMs

Very few studies have been conducted on triglycerides as possible PCMs for latent heat storage applications. However, organic materials such as esters and fatty acids have recently gained the interest of researchers owing to their low toxicity, sustainability and availability from renewable feedstock [44,50,51]. Nevertheless, compared to other classes of organic materials triglycerides have been overlooked for energy applications due to their complex behaviour in terms of polymorphism which can lead to unreliable results when not controlled properly.

Generally, as shown in the previous pages and tables, triglycerides cover a wide range of temperatures from the subzero region up to approximately 90 ${}^{\circ }$C. In particular, SST and SAT present higher enthalpies of fusion and higher thermal stabilities compared to UST. Many SST in their β phase are characterized by attractive phase change enthalpies, such as for example trilignocerin or tristearin with Δ*H* ≥ 190 J/g. In this regards, several SST reported in this study could prove to be interesting candidates as PCM for mid temperature applications (50 to 100 ${}^{\circ }$C). Some examples are the storage of excess heat, the provision of domestic hot water and the waste heat recovery in thermal batch processes in the food and beverage industry to improve the energy efficiency [67,68,69]. In fact, only few bio-based, non-flammable, non-toxic and sustainable organic materials with Tm ≥ 80 ${}^{\circ }$C with high energy densities have been tested for such applications, and they generally comprise of carboxylic acids or sugar alcohols, which present the disadvantage of being more reactive and prone to corrosivity than triglycerides [68].

The main materials used for mid temperature applications at the moment are salt hydrates, which present the advantage of possessing very high Δ*H* (generally between 150–250 J/g). As can be seen in (Table 1), this is comparable to the Δ*H* of SST. Despite being more established as PCMs, salt hydrates still present several disadvantages such as a high tendency to supercool, and a low long-term stability due to segregation. Both phenomena are still poorly understood and require extensive research to be solved. Additionally, most salt hydrates are either toxic or irritant, therefore can encounter limitations in their applicability [5,6].

Most organic PCMs used consist of paraffins [70,71]. Unlike salt hydrates, paraffins present the advantage of possessing low supercooling and being thermally reliable over consecutive heating-cooling cycles. Still, paraffins are derived from petroleum and are highly flammable. In addition, they present lower Δ*H* ≤ 200 J/g.

Triglycerides are sustainable and bio-based, possess higher Δ*H* than paraffins, and do not appear to undergo segregation. Therefore, they present considerable advantages compared to commonly- used PCMs.

To date, some studies have been conducted on readily available triglyceride-rich substances such as olive oil. The main fatty acid constituents of the triglycerides in olive oil are palmitic, palmitoleic, stearic, oleic, linoleic and linolenic acids, comprising of about 94–96% of the total weight of the triglycerides. However, it must be kept in consideration that the composition of olive oil is strongly dependent on the region of extraction and the growth conditions. Gunasekara et al. [72] measured the thermal properties and observed a consistent melting and freezing for four cycles at the temperatures −4.5 to 10.4 ${}^{\circ }$C and −8 to −11.9 ${}^{\circ }$C, with the enthalpies 105 and 97 kJ/kg respectively. While the enthalpies are too low to be considered suitable for LHS applications, they are in agreement with what has been reported previously, according to which the enthalpy of triglycerides mixtures is lower than that of simple triglycerides. Therefore, it might be difficult to evaluate the suitability of olive oil as PCM due to its complex composition and diversity.

One of the most relevant studies was conducted by Sari et al. [40], where the authors synthesized trimyristin, tripalmitin and tristearin with high purities and characterized their thermal properties. The authors also conducted long-term cycling tests by cycling the samples 1000 times in order to prove their thermal stability and reliability. While they did not attribute the melting points registered to any specific polymorphic phase, the values reported (Table 1) suggest that the authors did not successfully reach the stable β phase for any of the triglycerides analyzed (trimyristin 58.5 ${}^{\circ }$C, tripalmitin 66.5 ${}^{\circ }$C, tristearin 73.0 ${}^{\circ }$C). Instead, they formed the α phase for trimyristin (32.0 ${}^{\circ }$C) and the β‘ for both tripalmitin (58.5 ${}^{\circ }$C) and tristearin (63.5 ${}^{\circ }$C). This is not surprising since they performed the heating-cooling tests with heating rates of 5 K/min, which are too fast to allow for the formation of β crystals. Nevertheless, in every case only one crystalline phase was observed and the differential scanning calorimetry (DSC) analysis results showed that the repeated 1000 thermal cycling did not cause any degradation in the chemical structure of the esters or any significant change in their thermal properties. In addition and as reported by Ravotti et al. [44,50] on fatty esters, close to no supercooling was observed.

Saturated triglycerides therefore present attractive enthalpies of fusion and a wide temperature range, with high temperatures achievable in the β phase, which proves to be interesting for a variety of applications. Although Sari et al. [40] proved that the α and β′ phases are stable upon consecutive heating-cooling cycles, achieving the thermodynamically stable β form would ensure even higher reproducibility and energy densities. This would be possible for applications requiring extremely slow heating and cooling rates. Alternatively, passive seeding has proven to be a valuable option according to Suppes et al. [25]. Several ways to perform passive seeding exist. Alfutimie et al. [36] found that the addition of small percentages of monoglycerides favour the crystallization rate and the stabilization of the β phase. Another way to promote the β form is to add longer-chain triglycerides as nucleating agents, similarly to what is done with nucleating agents in salt hydrates to reduce the supercooling [73].

Nevertheless, the control of triglycerides’ polymorphism and the reproducible formation of desired phases over consecutive heating-cooling cycles are still far from being mastered.

One problem encountered by triglycerides and other organic materials is represented by the low thermal conductivities, generally around 0.2 W/(m·K), which together with the higher prices present challenges for the introduction of such materials in energy-related fields. However, several studies on the enhancement of the thermal conductivities of organic PCMs have been conducted. Most consist of including various amounts of nanographite (generally ≤10%) to improve the conductivity [40,74,75,76,77], while alternative methods use improved heat exchangers or foams [78,79,80,81]. In particular, Lazzarin et al. [78] achieved eight times faster loading and unloading times when using aluminum foams as heat transfer media, while several simulations and experimental setups comprising of finned-tube heat exchangers show promising results in terms of high power and heat conductivity [79,80,81].

## 6. Conclusions and Outlook

Triglycerides appear to possess interesting thermal properties suitable for LHS applications, with high enthalpies of fusion of up to approximately 200 J/g and a wide range of phase-change temperatures. Even-numbered saturated symmetrical triglycerides have the highest melting points and enthalpies of fusion due to a high degree of symmetry and to the absence of double bonds, which lower the compactness of the crystal packing and give rise to lower stabilities. This makes them interesting candidates for a broad range of applications, from sub-zero temperatures up to 90 ${}^{\circ }$C. In particular, triglycerides could prove to be valuable candidates as PCM for the higher temperature range for industrial heating applications, where no other bio-based, sustainable and renewable feedstock-derived organic substances are currently used.

In terms of thermal stability and reproducibility, although triglycerides have proved to be stable for several cycles and up to temperatures of 150 ${}^{\circ }$C or above without showing any signs of degradation, it is of utmost importance to achieve complete control over the formation of the desired polymorphic phases.

Although it has been shown in the literature that the reproducible formation of the desired polymorph directly from the melt is possible, either in controlled heating-cooling conditions or via addition of nucleating agents, further investigation need to be performed to evaluate the reproducibility and stability of the proposed methods.

In conclusion, triglycerides and especially saturated symmetrical triglycerides can prove to be a valuable class of materials for LHS applications on condition that the stable polymorphic β phase is obtained or induced. While some investigations on different methods to achieve this have started, such issue is still far from being solved. Besides the thermal energy storage world, the complete control over the formation of polymorphs from the melt would also greatly benefit the food industry.

## Figures and Tables

**Table 1:** Data collected on the melting points and enthalpies of fusion of saturated symmetrical triglycerides for all polymorphis. The abbreviation indicates the chain length of the precursor fatty acid; for example, tricaproin is derived from glycerol and three caproic acid molecules, of which the carbon number is 6. Therefore, tricaproin is indicated as 6.6.6. Next to the abbreviations, the common given names of each triglycerides are indicated. The melting points reported are based on the peak temperatures in ${}^{\circ }$C, and the enthalpies are expressed in J/g. The superscript numbers on each measurement value indicate the reference from which the value has been extracted. Numbers in brackets next to the main value indicate the specific type of “submodification” to which the value has been assigned by the author. For instance, the value 28 (1) for the β′ phase of tridecanoin indicate that the β′1 polymorph of tridecanoin melts at 28 ${}^{\circ }$C. Missing values have been indicated with the “-” sign.

Saturated Symmetrical Triglycerides (SST)							
**Structure**		**Melting Point (Peak [${}^{\circ }$C])**			**ΔH [J/g]**		
**Abbreviation**	**Given Name**	α	β′	β	α	β′	β
6.6.6	tricaproin	-	-	−25 [29]	-	-	-
7.7.7	trienanthin	-	-	-	-	-	-
8.8.8	tricaprylin	−51 [15], −54 [18]	−18 [15], −19 [18]	9.4 [8], 9.9 [15], 11 [18], 10 [29]	36.4 [18]	-	-
9.9.9	tripelargonin	−26 [15], −30 [18]	7 [18]	9.5 [8], 10.7 [15], 10 [18]	69.4 [18]	96.3 [18]	106.9 [18]
10.10.10	tricaprin	−11.5 [28], −9 [15], −10 [18], −15 [11]	16.9 [28], 15.5 [15], 13 [18] (3) [18], 18 (2) [18], 18 [11]	30.4 [8], 31.7 [28], 31.9 [15], 32.3 [15], 33 [18], 31.5 [11], 31.7 [29], 32 [41]	103.3 [28], 101.8 [18]	-	171.2 [28], 165.9 [18]
11.11.11	triundecanoin	3.7 [15], 4 [18], 1 [11]	27.5 [15], 28 (1) [18], 26.5 [11]	31.2 [15], 31 [18], 31.2 [41]	92.5 [18]	119.9 [18]	141.6 [18]
12.12.12	trilaurin	15.7 [28], 15.2 [15], 14 [18], 15 [11]	35.2 [28], 34 [15], 34 (2) [18], 35 [11]	46.3 [8], 45.8 [28], 46.5 [15], 46 [18], 46.4 [11], 46.5 [29], 46.4 [41]	109.2 [28], 112.6 [18]	134.6 [28]	191.2 [28], 182.0 [18]
13.13.13	tritridecanoin	24.4 [15], 23 [18], 25 [11]	41.5 [15], 40 (1) [18], 41 [11]	44.6 [8], 45.1 [15], 42 [18], 44 [11], 44.5 [41]	109.3 [18]	122.9 [18]	146.2 [18]
14.14.14	trimyristin	32.7 [28], 32.8 [15], 31 [18], 32.0 [40], 33 [11]	46 [28], 45 (2) [18], 46.5 [11]	57.4 [8], 57.2 [28], 58.5, 56 [18], 57 [11], 58 [29], 58.5 [41]	113.3 [28], 117.5 [18], 154.3 [40]	146.6 [28]	203 [28], 189.2 [18]
15.15.15	tripentadecanoin	38.5 [15], 41 [18], 40 [11]	50.1 [15], 54 (2) [18], 56 (1) [18], 51.5 [11]	55.5 [8], 56.2 [15], 58 [18], 54.6 [11]	126.8 [18]	142.2 [18]	188.6 [18]
16.16.16	tripalmitin	44.8 [28], 44.7 [15], 46 [18], 45 [11]	55.8 [28], 56.6 [15], 57 (1) [18], 58.5 [40], 56 [11]	66 [28], 66.4 [15], 66 [18], 65.5 [11], 66 [29], 65.4 [8], 66.4 [41]	118.7 [28], 127.5 [18]	156.7 [28], 185.9 [40]	212.2 [28], 205.2 [18], 260.7 [14]
17.17.17	trimargarin	50 [15], 50 [18], 50 [11]	60.1 [15], 60 (2) [18], 61 [11]	64 [18], 64.5 [15], 65 [18], 64 [11], 64 [41]	129.1 [18]	151.2 [18]	199.0 [18]
18.18.18	tristearin	54.8 [28], 54.7 [15], 55 [18], 54.5 [11]	64.4 [28], 63.2 [15], 64 (1) [18], 63.5 [40], 65 [11]	72.7 [8], 72.6 [28], 73.5 [15], 73 [18], 72 [11], 72.6 [29], 73 [41]	121.7 [28], 125.8 [18]	175.5 [28], 184.9 [14], 149.4 [40]	217.8 [28], 215 [18], 257.7 [14]
19.19.19	trinonadecanoin	58.2 [15], 60 [18]	64.5 [15], 65 (2) [18]	71.3 [8], 71.4 [15], 72 [18], 71 [41]	129.1 [18]	145.7 [18]	199.9 [18]
20.20.20	triarachidin	6328, 61.8 [15], 64 [18]	69.6 [28], 69 [15], 69 (2) [18]	77.7 [8], 77.9 [28], 78.1 [15], 78 [18], 78.1 [29], 78 [41]	126.6 [28], 125.2 [18]	164.4 [28]	219.5 [28], 226 [18]
21.21.21	triheneicosanoin	65 [15], 66 [18]	71 [15], 70 (2) [18], 73 (1) [18]	76.4 [8], 75.9 [15], 76 (2) [18], 77 (1) [18], 75.9 [41]	125.4 [18]	147.2 [18]	177.6 [18]
22.22.22	tribehenin	68.8 [28], 68.2 [15], 69 (2) [18], 70 (1) [18]	74.92 [8], 74 [15], 74 (2) [18]	82.5 [8], 81.8 [28], 82.5 [15], 83 [18], 82.5 [29], 82.5 [41]	135.1 [28], 135 [18]	147.9 [28], 143.7 [18]	213.7 [28], 211.2 [18]
23.23.23	tritricosanoin	71 (2) [18], 72 (1) [18]	75 (2) [18], 76 (1) [18]	81.9 [8], 80 (2) [18], 81 (1)1 [18]	127.6 [18]	144.7 [18]	173.5 [18]
24.24.24	trilignocerin	74 (2) [18], 75 (1) [18]	79 (2) [18], 81 (1) [18]	86 [18], 86 [29], 86 [41]	140.4 [18]	136.1 [18]	197.9 [18]
25.25.25	tripentacosanoin	-	-	-	-	-	-
26.26.26	tricerotin	78 (2) [18], 79 (1) [18]	82 (2) [18], 85 (1) [18]	89 [18]	132.2 [18]	129.8 [18]	190.8 [18]
27.27.27	tricarbocerin	-	-	-	-	-	-
28.28.28	trimontanin	80 (2) [18], 82 (1) [18]	91 (1) [18]	-	86.7 [18]	132.3 [18]	-
29.29.29	trinonacosanoin	-	-	-	-	-	-
30.30.30	trimelissin	79 (2) [18], 83 (1) [18]	93 (1) [18]	-	68.9 [18]	107.5 [18]	-

**Table 2:** Data collected on the melting points and enthalpies of fusion of unsaturated symmetrical triglycerides for all polymorphs. The abbreviation indicates the chain length of the precursor fatty acid and the unsaturation position in the aliphatic chain; for example, tripalmitolein is derived from glycerol and three palmitoleic acid molecules, whose carbon number is 16 and that have 1 double bond in configuration cis at the carbon number 9. Therefore, tripalmitolein is indicated as 16.16.16:1 cis 9. Next to the abbreviations, the common given names of each triglycerides are indicated. The melting points reported are based on the peak temperatures in ${}^{\circ }$C, and the enthalpies are expressed in J/g. The superscript numbers on each measurement value indicate the reference from which the value has been extracted. Numbers in brackets next to the main value indicate the specific type of “submodification” to which the value has been assigned by the author. For instance, the values −12 (3), −8 (2) and −5 (1)for the β′ phases of triolein indicate that the β′1, β′2 and β′1 polymorphs of triolein melt at −5, −8 and −12 ${}^{\circ }$C respectively. Missing values have been indicated with the “-” sign.

Unsaturated Symmetrical Triglycerides (UST)							
**Structure**		**Melting Point (Peak [${}^{\circ }$C])**			**ΔH [J/g]**		
**Abbreviation**	**Given Name**	α	β′	β	α	β′	**β**
14.14.14:1 cis 9	Trimyristolein	-	-	-	-	-	-
16.16.16:1 cis 9	Tripalmitolein	−25.8 [41]	−22.8 [41], −21.9 [41], −21.8 [41]	25.7 [41]	-	-	-
16.16.16:1 trans 9	Tri-*trans*-9-hexadecenoin	-	-	-	-	-	-
16.16.16:1 cis 6	(6Z)-trihexadecenoin	−15.0 [16]	-	15.0 [16]	-	-	115.9 [16]
18.18.18:1 cis 6	Tripetroselinin	−2.0 (estimated) [16]	-	28.0 [8,16]	-	-	128.4 [16]
18:18:18:1 trans 6	Tripetroselaidin	32.0 (estimated) [16]	-	52.0 [8,16]	-	-	179.9 [16]
18.18.18:1 cis 9	Triolein	−32.0 [11], −37.0 [16], −32.0 [8]	−12.0 [11], −12.0 (3) [16], −8.0 (2) [16], −5.0 (1) [16], −5.5 [8]	−2.5 (onset) [17], 4.9 [14], 5.0 [16], 5.0 [8], 4.0 [41], 5.0 [45]	-	-	105.9 [17], 108 [16]
18.18.18:1 trans 9	Trielaidin	15.5 [11], 15.0 [16]	37.0 [11]	41.5, 41.0 [16]	-	-	165.3 [16]
18.18.18:1 cis 11	Tri-*cis*-11-octadecenoin	−11.0 (estimated) [16]	−3.0 (3) [16], 0.0 (2) [16], 3.0 (1) [16], 1.0 [41]	10.0 [16]	-	105.9 [16]	113.4 [16]
18.18.18:1 trans 11	Trivaccenin	-	-	-	-	-	-
18.18.18:2 cis 9-12	Trilinolein	−43.0 [11], −45.6 [31]	-	−12.9 [11], −12.9 [31], −11.0 [8], −12.7 [41]	-	-	-
18.18.18:3 cis 9-12-15	tri-α-linolenin	−84.0 [16]	−44.6 [31], −47.0 (3) [16], −27.0(2) [16], −21.0 (1) [16]	−24.2 [31]	-	-	96.2 [31]
19.19.19:1 cis 10	(10Z)-trinonadecenoin	26.1 [41] (no mention)			-	-	-
20.20.20:1 cis 9	Trigadolein	-	-	-	-	-	-
20.20.20:1 cis 11	Trieicosenoin	10.1 [41], 17.8 [41] (no mention)			-	-	-
21.21.21:1 cis 12	(12Z)-trihenecosenoin	38.0 [41](no mention)			-	-	-
22.22.22:1 cis 13	Trierucin	6.0 [11], 12.0 (estimated) [16]	25.0 [11], 22.0 (3) [16], 25.0 (2) [16], 29.0 (1) [16], 29.8 [41]	32.5 [11], 32.0 [16], 32.0 [8], 32.0 [41]	-	-	138.1 [16]
22.22.22:1 trans 13	(13E)-tridocosenoin	40.0 [16]	50.0 (2) [16], 56.0 (1) [16]	58.0 [16], 58.0 [8], 58 [41]	-	-	143.9 [16]
24.24.24:1 cis 15	Trinervonin	-	-	-	-	-	-

**Table 3:** Data collected on the melting points and enthalpies of fusion of saturated asymmetrical triglycerides for all polymorphs. The abbreviation indicates the chain length of the precursor fatty acid: for example, 1,3-dilaurin-2-caprin is derived from glycerol, two lauric acid molecules and one capric acid molecule, whose carbon numbers are 12, 12 and 10 respectively. The two lauric acid chains are found at the extremes of the molecule (positions 1 and 3), while the capric acid is in the middle (position 2). Therefore, 1,3-dilaurin-2-caprin is indicated as 12.10.12. Next to the abbreviations, the common given names of each triglycerides are indicated with a short abbreviation within brackets. The abbreviations are written by selecting the first letter of the precursor fatty acid in the position in which is found in the molecule. In particular: L = lauric acid, C = capric acid, M = myristic acid, P = palmitic acid, S = stearic acid, B = behenic acid. Additionally, SAT have been divided in different subgroups based on the acid of provenance. For example, LCL, PMP and SPS are all derived from two acids with a certain carbon number at the extremes of the molecule (n), and an acid shorter than the others by 2 carbons (n-2) in the middle position. As such, they belong to the group n.n-2.n. The group n1.n2.n3 includes triglycerides derived from three different fatty acids. The melting points reported are based on the peak temperatures in ${}^{\circ }$C, and the enthalpies are expressed in J/g. The superscript numbers on each measurement value indicate the reference from which the value has been extracted. Missing values have been indicated with the “-” sign.

Saturated Asymmetrical Triglycerides (SAT)							
**Structure**		**Melting Point (Peak [${}^{\circ }$C])**			**ΔH [J/g]**		
**Abbreviation**	**Given Name**	α	β′	β	α	β′	β
n.n-2.n							
12.10.12	1,3-dilaurin-2-caprin (LCL)	5.1 [28], 2.0 [11]	33.1 [28], 33.0 [11]	37.5 [28], 38.5 [11], 38.5 [29]	-	-	-
16.14.16	1,3-dipalmitin-2-myristin (PMP)	39.2 [28]	51.1 [28]	60.0 [28], 60.0 [29]	101.4 [28]	-	175.8 [28]
18.16.18	1,3-distearin-2-palmitin (SPS)	50.8 [28], 50.0 [11]	64.1 [28]	68.1 [28], 68.0 [11], 68.0 [29]	119.3 [28]	-	197.2 [28]
n.n+2.n							
10.12.10	1,3-dicaprin-2-laurin (CLC)	6.1 [28], 6.0 [11]	37.8 [28], 34.0 [11]	37.6 [28], 37.5 [11]	117.8 [28]	153.8 [28]	-
14.16.14	1,3-dimyristin-2-palmitin (MPM)	36.3 [28], 37.0 [11]	59.6 [28], 55.0 [11]	59.3 [28], 60.0 [11]	124.0 [28]	169.3 [28]	-
16.18.16	1,3-dipalmitin-2-stearin (PSP)	47.3 [28], 49.0 [11]	67.8 [28], 65.0 [11], 65.5 [29]	68.0 [11]	134.3 [28]	198.1 [28]	-
n.n.n+2							
10.10.12	1,2-dicaprin-3-laurin (CCL)	0.1 [28], 0.0 [11]	26.1 [28], 26.0 [11]	30.1 [28], 30.0 [11], 30.0 [29]	-	-	-
14.14.16	1,2-dimyristin-3-palmitin (MMP)	26.9 [28], 36.0 [11]	48.6 [28], 47.5 [11]	53.4 [28], 52.0 [11], 54.0 [29]	108.0 [28]	133.3 [28]	174.6 [28]
16.16.18	1,2-dipalmitin-3-stearin (PPS)	46.5 [28], 46.5 [11]	58.8 [28], 59.5 [11]	62.7 [28], 62.5 [11], 62.5 [29]	119.6 [28]	148.3 [28]	198.9 [28]
n.n+4.n+4							
10.14.14	1-caprin-2,3-dimyristin (CMM)	15.1 [28], 15.0 [11]	38.1 [28], 38.0 [11]	43.6 [28], 43.5 [11], 43.5 [29]	-	-	-
12.16.16	1-laurin-2,3-dipalmitin (LPP)	32.1 [28]	49.6 [28]	54.5 [28], 54.0 [29]	-	-	-
18.22.22	1-stearin-2,3-dibehenin (SBB)	61.4 [28]	71.6 [28]	73.6 [28], 73.2 [29]	-	-	-
n1.n2.n3							
18.10.16	1-stearin-2-caprin-3-palmitin (SCP)	20.2 [28]	53.9 [28]	54.3 [28]	106.8 [28]	-	166.8 [28]
16.14.12	1-palmitin-2-myristin-3-laurin (PML)	36.6 [28]	44.1 [28]	48.6 [28]	102.3 [28]	130.0 [28]	172.9 [28]
18.14.16	1-stearin-2-myristin-3-palmitin (SMP)	41.0 [28]	56.2 [28]	59.7 [28]	115.2 [28]	-	188.3 [28]

**Table 4:** Data collected on the heat capacities [J/(g·K)] and thermal conductivities [W/(m·K)] of some saturated symmetrical triglycerides. The abbreviation indicates the chain length of the precursor fatty acid; for example, tricaproin is derived from glycerol and three caproic acid molecules, whose carbon number is 6. Therefore, tricaproin is indicated as 6.6.6. Next to the abbreviations, the common given names of each triglycerides are indicated. The superscript numbers on each measurement value indicate the reference from which the value has been extracted. Missing values have been indicated with the “-“ sign.

Structure		Heat Capacity $C_{p}$ [J/(g·K)] (at Room Temperature)	Thermal Conductivity λ [W/(m·K)] (at Room Temperature)
Abbreviation	Given Name		
6.6.6	tricaproin	1.88 [47]	-
8.8.8	tricaprylin	1.94 [44,48], 1.96 [49]	0.15 [46]
10.10.10	tricaprin	1.97 [44,48], 2.08 [49]	0.15 [46]
12.12.12	trilaurin	2.06 [44,48], 2.05 [47]	0.16 [46]
14.14.14	trimyristin	2.14 [46,48], 2.10 [47]	0.17 [46], 0.23 [40]
16.16.16	tripalmitin	2.20 [47]	0.19 [40]
18.18.18	tristearin	2.23 [47]	0.17 [40]

**Figure A1:**
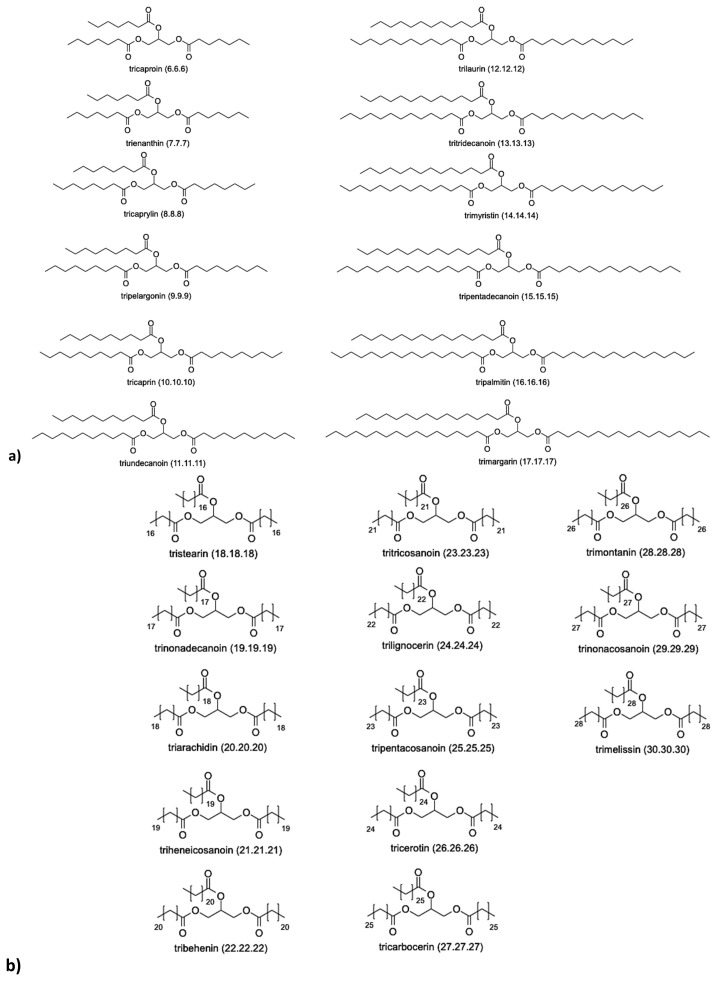
Chemical structures of Saturated Symmetrical Triglycerides (SST). (**a**) SST’s chemical structures from tricaproin (6.6.6) to trimargarin (17.17.17). (**b**) SST’s chemical structures from tristearin (18.18.18) to tricarbocerin (27.27.27). For visibility purposes, structures with carbon numbers higher than 18 in each chain have been abbreviated.

**Figure A2:**
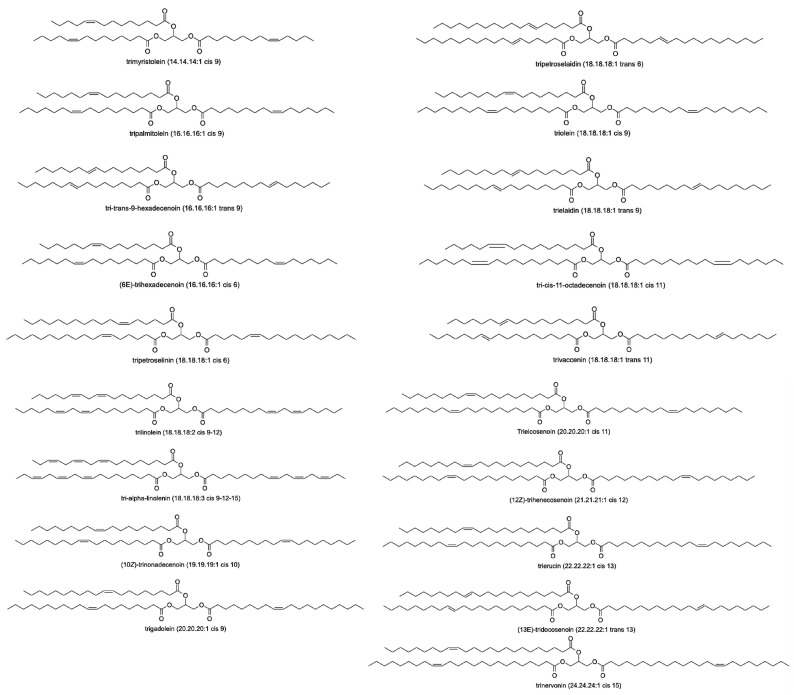
Chemical structures of Unsaturated Symmetrical Triglycerides (UST).

**Figure A3:**
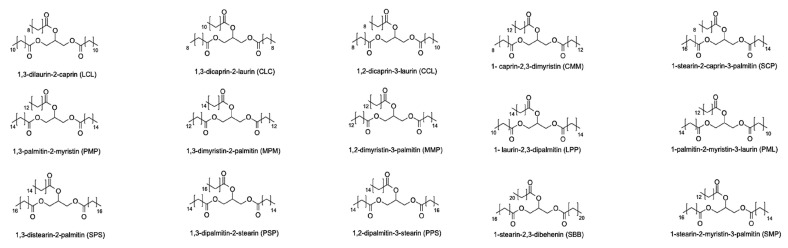
Chemical structures of Saturated Asymmetrical Triglycerides (SAT). For visibility purposes, the alkyl chains have been abbreviated.

## Abbreviations

The following abbreviations are used in this manuscript:

CCL	1,2-dicaprin-3-laurin
CLC	1,3-dicaprin-2-laurin
CMM	1-caprin-2,3-dimyristin
*$C_{p}$*	Specific heat [J/(g·K)]
DSC	Differential Scanning Calorimetry
GC method	Group contribution method
LCL	1,3-dilaurin-2-caprin
LHS	Latent Heat Storage
LPP	1-laurin-2,3-dipalmitin
MMP	1,2-dimyristin-3-palmitin
MPM	1,3-dimyristin-2-palmitin
PCM	Phase Change Material
PML	1-palmitin-2-myristin-3-laurin
PMP	1,3-dipalmitin-2-myristin
PPS	1,2-dipalmitin-3-stearin
PSP	1,3-dipalmitin-2-stearin
SAT	Saturated Asymmetrical Triglycerides
SBB	1-stearin-2,3-dibehenin
SCP	1-stearin-2-caprin-3-palmitin
SMP	1-stearin-2-myristin-3-palmitin
SNSF	Swiss National Science Foundation
SPS	1,3-distearin-2-palmitin
SST	Saturated Symmetrical Triglycerides
TES	Thermal Energy Storage
UST	Unsaturated Symmetrical Triglycerides
XRD	X-Ray Diffraction
α	Kinetically stable polymorphic phase
β′	Metastable polymorphic phase
β	Thermodynamically stable polymorphic phase
γ	Glass-like polymorphic phase, today intended same as α
λ	Thermal conductivity [W/(m·K)]
Δ*H*	Gravimetric enthalpy of fusion [J/g]

## Appendix A. Chemical Structures

In this Appendix, all the chemical structures of the triglycerides mentioned in the text are reported.
